# *Bangia fusco-purpurea* Vegan Sausages: Orthogonal Experimental Optimization and Gel Formation Mechanism

**DOI:** 10.3390/foods14173014

**Published:** 2025-08-28

**Authors:** Xiaoting Chen, Shiqing Zhuo, Nan Pan, Yongchang Su, Zhiyu Liu, Jingna Wu

**Affiliations:** 1 Key Laboratory of Cultivation and High-Value Utilization of Marine Organisms in Fujian Province, National Research and Development Center for Marine Fish Processing (Xiamen), Fisheries Research Institute of Fujian, No. 7, Haishan Road, Huli District, Xiamen 361013, China; xtchen@jmu.edu.cn (X.C.); npan01@qub.ac.uk (N.P.); suyongchang@stu.hqu.edu.cn (Y.S.); 2 Xiamen Key Laboratory of Marine Medicinal Natural Products Resources, Fujian Universities and Colleges Engineering Research Center of Marine Biopharmaceutical Resources, Xiamen Medical College, No. 1999, Guankou Middle Road, Jimei District, Xiamen 361023, China; siny47@163.com

**Keywords:** vegan sausages, *Bangia fusco-purpurea*, process optimization, gluten, complex gel, physicochemical properties

## Abstract

To develop highly nutritious *Bangia fusco-purpurea* (BFP) vegan sausages, we investigated the effects of BFP, gluten, and xanthan gum–konjac gum–carrageenan complex gel (CG) on the gel strength and sensory quality of the sausages. The formulation process was optimized through single-factor and orthogonal tests, whereas the gel formation mechanism of the key factors was explored. The orthogonal test results showed that the optimal addition levels of BFP, gluten, and CG were 5%, 56%, and 37%, respectively. Variance analysis revealed that both gluten and CG significantly affected gel strength (*p* < 0.05), with gluten notably influencing the overall sensory quality (*p* < 0.05). Texture profile analysis (TPA) and rheological properties demonstrated that as gluten (33–37%) and CG (52–56%) concentrations increased, the gel strength and elastic modulus exhibited concentration-dependent enhancement. Further analysis of the sulfhydryl content, disulfide bonds, surface hydrophobicity, and microstructure revealed that higher gluten content promoted intermolecular disulfide crosslinking and hydrophobic group exposure, whereas CG contributed to physical filling via hydrogen and ionic bonds, resulting in a uniform and dense gel network structure. The synergistic effects of gluten and CG enhanced the gel properties of BFP vegan sausages, providing a theoretical foundation for the development of high-quality plant protein-based meat alternatives.

## 1. Introduction

The rising demand for animal meat has led to challenges such as greenhouse gas emissions and human health risks [1]. Plant-based meat analogs, manufactured from extracted plant proteins through extrusion, 3D printing, or mixing technologies, have gained significant attention as sustainable substitutes for animal meat [2]. This prominence stems not only from their texture and taste mimicry capabilities, but also from their reduced environmental footprint and alignment with ethical consumption trends [3]. However, conventional plant-based meats, primarily based on soy or wheat proteins, face nutritional limitations due to their singular composition and often lack essential nutrients such as iron and vitamin B12. *Bangia fusco-purpurea* (BFP), a high-value red seaweed rich in polysaccharides, dietary fiber, proteins, and natural pigments (e.g., phycoerythrin), offers both nutritional enhancement and functional benefits, and shows great potential for food applications [4,5,6]. Preliminary studies have confirmed that the BFP protein is abundant in essential amino acids and can offset the methionine and lysine deficiencies in cereal proteins when used in plant-based meat products [7]. In addition, its dietary fiber and trace elements (e.g., Fe, Zn, and Ca) provide natural nutritional advantages.

As an important category of plant-based meat, vegan sausages primarily target individuals aged ≥12 years, specifically designed for ethical vegans and health-focused consumers. The core of vegan sausage processing lies in constructing a superior gel network performance to form a gel matrix with a meat-mimetic fibrous structure. Current research primarily focuses on optimizing texture through synergistic interactions between plant proteins and hydrophilic colloids such as polysaccharides. Plant proteins provide structural support and elasticity, while polysaccharides enhance hydration and textural resilience [8]. Gluten (a wheat protein isolate) contributes to unique viscoelasticity by forming thermally induced *β*-sheet structures within its elastic gel network, improving the springiness and chewiness of the product. Meanwhile, polysaccharide colloid exhibited enhanced water-holding capacity via intermolecular hydrogen bonds and ionic crosslinking. For instance, transglutaminase (TGase)-catalyzed ε-(γ-glutamyl)lysine crosslinking combined with deacetylated konjac glucomannan can increase gel strength by 2.3-fold while elevating water retention from 75% to 89% [9,10]. Similarly, sulfated polysaccharide–hydrogen bonding interactions with pea protein emulsions boost gel hardness by 75-fold, along with a 37% improvement in water retention [11]. However, the introduction of high-fiber red seaweed may disrupt protein–hydrocolloid synergy. Moreover, excessive addition may cause off-flavors and color abnormalities. Consequently, balancing the functionality of red seaweed with sensory quality through formulation optimization is a critical challenge in developing high-performance vegan sausages.

Therefore, this study aimed to optimize the formulation processing of BFP-based vegan sausages using a single-factor experiment and orthogonal methodology, while identifying the effects of key factors (BFP, gluten, and CG) on the gel strength during gel formation. Additionally, this study sought to reveal the gelation regulatory mechanisms of protein molecular conformational transition patterns by measuring changes in free sulfhydryl (FS), total sulfhydryl (TS) groups, disulfide bonds (S-S bonds), and surface hydrophobicity (SH). Through analyzing texture characteristics, microstructural networks, and dynamic rheological behavior of vegan sausage gels from multiscale perspectives, this study provides a theoretical foundation for precise texture regulation in plant-based meat products.

## 2. Materials and Methods

### 2.1. Materials and Reagents

BFP was purchased from Putian Hai Dao Ren Jia Aquatic Products Co., Ltd. (Putian, China). Soybean protein isolate (SPI) was purchased from Guangzhou Huaxi Bioengineering Co., Ltd. (Guangdong, China). Gluten and compound phosphates were purchased from Fengqiu Huafeng Starch Co., Ltd. (Henan, China). Corn starch was purchased from Foshan Zongwang Food Co., Ltd. (Guangdong, China). Xanthan gum was purchased from Xinjiang Meihua Amino Acid Co., Ltd. (Xinjiang, China). Konjac gum was purchased from Hubei Qiangsen Konjac Technology Co., Ltd. (Hubei, China). Carrageenan was purchased from Zhaoqing Haitian Biotechnology Co., Ltd. (Guangdong, China). Sugar was purchased from Foshan Chengcheng High Food Co., Ltd. (Guangdong, China). Salt was purchased from China National Salt Industry Co., Ltd. (Beijing, China). Yeast extract was purchased from Angel Yeast Co., Ltd. (Hubei, China). Disodium 5′-Inosinate and Disodium 5′-Guanylate (I + G) were purchased from Henan Wansheng Food Technology Co., Ltd. (Hunan, China). Soybean oil was purchased from Arawana Grain and Oil Industry Co., Ltd. (Guangdong, China). Synthetic casings were purchased from Tianjin Kangtai Synthetic Packaging Co., Ltd. (Tianjin, China). Phosphate-buffered saline (PBS) was purchased from Wuhan Service Bio Co., Ltd. (Beijing, China). Glycine was purchased from Beijing Solarbio Science and Technology Co., Ltd. (Beijing, China). Tris was purchased from Sangon Biotech (Shanghai) Co., Ltd. (Shanghai, China). The 5,5′-dithiobis-(2-nitrobenzoic acid) (DTNB) solution was purchased from Shanghai YuanYe Biotechnology Co., Ltd. (Shanghai, China). Bromophenol blue, anhydrous ethanol, trichloroacetic acid, and hydrochloric acid were purchased from Shanghai HuShi Environmental Reagent Technology Co., Ltd. (Shanghai, China). Phosphate-buffered saline (PBS) was purchased from Wuhan Service Bio Co., Ltd. (Beijing, China). Glycine (≥99.9%) was purchased from Beijing Solarbio Science and Technology Co., Ltd. Tris (≥99.9%) was purchased from Sangon Biotech (Shanghai) Co., Ltd. (Shanghai, China). The 5,5′-Dithiobis-(2-nitrobenzoic acid) solution (DTNB, ≥98%) solution was purchased from Shanghai YuanYe Biotechnology Co., Ltd. (Shanghai, China). Bromophenol blue (≥98%), anhydrous ethanol (≥99%), trichloroacetic acid (≥99%), and hydrochloric acid (36–38%, GR grade) were purchased from Shanghai HuShi Environmental Reagent Technology Co., Ltd. (Shanghai, China).

### 2.2. Preparation of BFP Vegan Sausages

**Figure 1:**
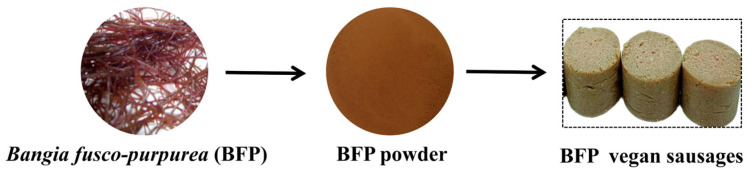
Images of *Bangia fusco-purpurea* (BFP) and BFP vegan sausages.

BFP was harvested from the coastal region of Putian city, Fujian Province, China, immediately frozen on ice, and then transported to the laboratory for storage at −20 °C. Using the laboratory’s preliminary research methods [12], the BFP was freeze-dried under vacuum, ground into powder, and passed through a 1000 mesh sieve to prepare the BFP powder (Figure 1). Firstly, SPI, CG (xanthan gum: konjac gum/carrageenan = 1:1:1, m:m:m), corn starch, gluten, and a portion of ice water were added to a bowl cutter (SJJ-E08G1, Bear Electric Appliance Co., Ltd., Guangdong, China), then subjected to high-speed chopping for 30 min. Subsequently, BFP powder, white sugar, salt, compound phosphates, yeast extract, I + G, and the remaining ice water were added, followed by an additional 30 min of high-speed chopping. Finally, soybean oil was added, and the mixture was chopped at high speed for 30 min to achieve homogeneity. The resulting mixture was stuffed into synthetic casings (24 mm in diameter × 12 cm in length) and hermetically sealed at both ends. The stuffed sausages were pre-heated in a water bath at 45 °C for 20 min, boiled at 95 ± 2 °C for 1 h, and then immediately cooled in ice water to room temperature. The products (Figure 1) were refrigerated at 4 °C for 12 h and stored at 4 °C for 24 h before analysis.

### 2.3. Design of Single-Factor and Orthogonal Experiments

The effects of additive ingredients of BFP, CG, gluten, and water on the gel strength were evaluated using single-factor experiments (Table 1). Based on these results, two additional levels near the optimal range were selected for BFP, CG, and gluten, with narrow concentration ranges [13,14]. The final optimization was performed using an L_9_(3^4^) orthogonal array design with gel strength and sensory scores as evaluation criteria, following standard industrial practices for efficiently optimizing food formulations through minimal experimental runs while maintaining statistical reliability (Table 2).

### 2.4. Sensory Evaluation

A sensory evaluation panel comprising ten food science graduate students (five males and five females, aged 25–30) was established. Prior to formal evaluation, panelists underwent a two-week professional training program covering sensory evaluation principles, methodologies, and scoring criteria to ensure accurate attribute recognition. Their sensory capabilities, consistency, and reliability were assessed to confirm compliance with fundamental requirements. The study was approved by the Ethics Committee (FRIF21-2507-01), and participants provided written informed consent. Samples were scored on a 10-point scale (0: extremely poor, 10: excellent) for color, hardness, elasticity, and cross-section morphology, as detailed in Table 3. During evaluations, vegan sausages were reheated in boiling water for 2 min, randomly served on white porcelain dishes at room temperature under natural light, with water provided for palate cleansing.

### 2.5. Gel Strength Determination

The gel strength of the vegan sausage was determined using a TA-XT Plus texture analyzer (Stable Micro Systems, Godalming, UK) equipped with a P/5S probe (5 mm spherical head plunger). Vegan sausage samples (24 mm thick) were cut and conditioned at room temperature. The test parameters were as follows: pre-test speed of 60 mm/min; test speed of 60 mm/min; post-test speed of 60 mm/min; trigger force of 5 g; and 50% strain.

### 2.6. Texture Profile Analysis (TPA)

The texture profile of the vegan sausage was determined according to the methodology proposed by Carhuancho-Colca et al. (2024) [15] with some modifications. Vegan sausage samples (24 mm thick) were cut and conditioned at room temperature. Elasticity, cohesiveness, springiness, and chewiness were determined using a TA-XT Plus texture analyzer (Stable Micro Systems, Godalming, UK) equipped with a P/36R cylindrical probe. The test parameters were as follows: compression mode, pre-test speed of 2.0 mm/s, test speed of 1.0 mm/s, post-test speed of 1.0 mm/s, trigger force of 5 g, and 50% strain.

### 2.7. Rheological Properties Determination

The rheological properties of the vegan sausage were measured using a Haake Mars40 rheometer (Thermo Fisher Scientific, Bremen, Germany) with a 50 mm diameter plate. The samples were then crushed and evenly spread on the testing platform. The test mode was set to a temperature scan with an oscillation frequency of 0.1 Hz, plate spacing of 1 mm, strain of 1.0%, heating range of 45–100 °C, and heating rate of 2.0 °C/min. The changes in the storage modulus (G′) and loss modulus (G′) during heating were then recorded.

### 2.8. UV-Vis Spectral Analysis

The UV absorption spectra of the vegan sausage were obtained using an SP-752 UV-Vis spectrophotometer (Shanghai Yuanxi Analytical Instruments Co., Ltd., Shanghai, China). 1 g sample was immersed in 15 mL of distilled water for 30 min, and the supernatant obtained after phase separation was scanned from 200 to 400 nm.

### 2.9. Scanning Electron Microscopy (SEM)

The microstructures of the vegan sausage were determined using a FLex SEM 1000 II scanning electron microscope (Hitachi, Ltd., Tokyo, Japan) according to Xu et al. (2022) [16] with slight modifications. After lyophilization, the vegan sausage samples were sectioned into 2 × 2 × 2 mm thin slices. These slices were subsequently sputter-coated with gold and examined using SEM at a magnification of 400× and an accelerating voltage of 5 kV.

### 2.10. Free Sulfhydryl (FS) Groups Determination

The FS groups in the vegan sausage were determined following the method described by Liu et al. [17]. Vegan sausage (60 mg) was dispersed in 1 mL of PBS, then 5 mL of Tris-Gly buffer was added. The mixture was shaken, and 50 µL of Ellman’s reagent was added. The mixture was then incubated in the dark for 1 h. After incubation, the mixture was centrifuged at 3000 rpm for 10 min. A control sample without Ellman’s reagent was prepared using the same procedure. The absorbance of the supernatant of each sample was measured at 412 nm using a spectrophotometer. Equation (1) was used to calculate FS, where 73.53 is the molar extinction coefficient of Ellman’s reagent (mol/mL), A_412_ is the absorbance at 412 nm, C is the dilution factor, and M is the protein mass concentration (mg/mL).

(1) $$FS/(mol/g)=73.53\;\times \;A_{412}\;\times \;C/M$$

### 2.11. Protein Mass Concentration Determination

The protein mass concentration was determined by the biuret method. 3 g of vegan sausage sample was added to 15 mL of cold high salt buffer solution (0.6 M NaCl, 20 mM Tris-HCl, pH 7.0). The mixture was homogenized for 2 min and then centrifugation (4 °C, 15,000× *g*, and 15 min). Protein content in the obtained supernatant was determined by the biuret method using bovine serum albumin as a standard.

### 2.12. Total Sulfhydryl (TS) Groups and Disulfide Bonds (S-S Bonds) Determination

The determination of TS groups and S-S bonds in vegan sausage was performed according to the method outlined by Liu et al. [17]. Vegan sausage (120 mg) was dispersed in 2 mL of PBS. After adding 4 μL of *β*-mercaptoethanol, the mixture was incubated for 2 h. Then, 4 mL of 12% trichloroacetic acid solution was added, and the mixture was allowed to stand for 1 h. After centrifugation at 10,000 rpm for 20 min, the precipitate was washed thrice with a 12% trichloroacetic acid solution, with each wash followed by centrifugation at 10,000 rpm for 10 min. The supernatant was removed, and 2 mL of Tris-GLy buffer and 80 μL of Ellman’s reagent were added. After vigorous shaking, the mixture was left to stand at 25 °C for 1 h and then centrifuged at 10,000 rpm for 30 min. The absorbance of the supernatant was measured at 412 nm using a control sample without Ellman’s reagent. Each group was subjected to three parallel tests per group. The TS content was calculated using Equation (1), and the S-S bond content was calculated using Equation (2).

(2) S-S bonds/(mol/g) = (TS − FS)/2

### 2.13. Surface Hydrophobicity (SH) Determination

The SH of the vegan sausage was determined by adhering to the protocol of Xing et al. (2024) [18] with minor changes. Vegan sausage (100 mg) was dispersed in 10 mL of PBS. Then, 80 µL of 1 mg/mL bromophenol blue solution was added and mixed thoroughly. The mixture was shaken at room temperature for 10 min and centrifuged at 5000 rpm for 15 min. The control group was prepared using PBS with 80 µL of bromophenol blue. The supernatant was collected, and the absorbance was measured at 595 nm. Three parallel experiments were performed for each treatment group. The calculation of SH based on bromophenol blue binding capacity is shown in Equation (3). Where A_0_ is the absorbance of the bromophenol blue-containing phosphate buffer, A_1_ is the absorbance of the sample supernatant, and m is the mass of bromophenol blue added (80 µg).

(3) $$SH/(µg)\;=\;m\;\times \;(A_{0}\;-\;A)/A_{0}$$

### 2.14. Statistical Analysis

Data obtained in triplicate were expressed as mean ± standard deviation. Statistical analysis was performed using SPSS Statistics 21.0 software (IBM Corp. Armonk, NY, USA). One-way ANOVA analysis was conducted, and the significance of the observed main effects was determined using Duncan’s multiple comparison test (*p* < 0.05). Origin 2023 software (OriginLab, Northampton, MA, USA) was used for plotting.

## 3. Results and Discussion

### 3.1. Analysis of Single-Factor Experiment Results

**Figure 2:**
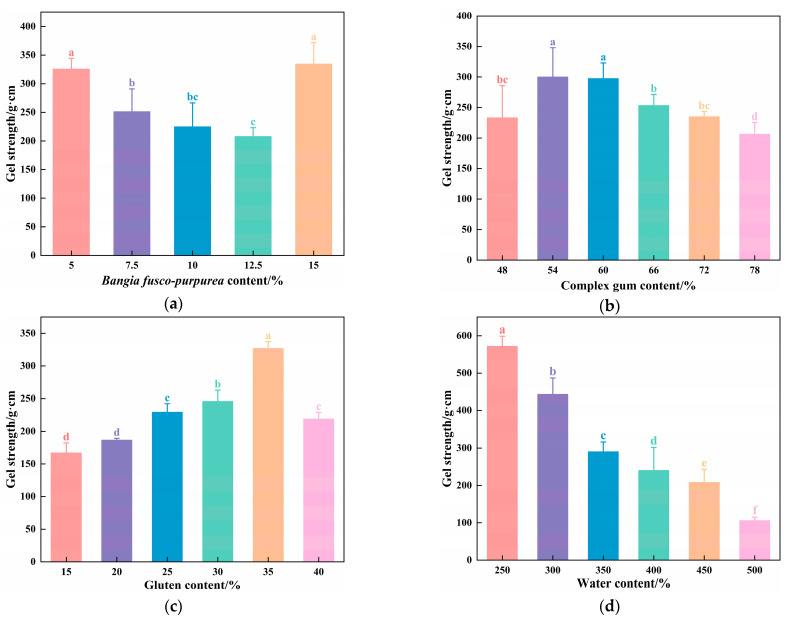
Effects of (**a**) BFP; (**b**) CG; (**c**) gluten; (**d**) water on the gel strength of BFP vegan sausages. Different letters indicate significant differences (*p* < 0.05).

As shown in Figure 2a, the gel strength of the vegan sausages initially decreased and then increased as the BFP content increased from 5% to 15%. The gel strength was maximized at 5% and 15% BFP content, reaching 325.49 ± 18.90 g·cm and 334.40 ± 37.18 g·cm, respectively, with no significant difference between them (*p* > 0.05). This could be attributed to the high polysaccharide content of the BFP. This could be attributed to the high polysaccharide content of the BFP. Interactions between these polysaccharides and proteins-such as hydrogen bonding, hydrophobic interactions, and electrostatic forces-significantly influence gel formation and stability. At a 5% BFP content, polysaccharide-protein co-crosslinking is enhanced, strengthening intermolecular hydrogen bonds within the protein matrix, which elevates solution viscosity and improves rheological properties, thereby reinforcing gel cohesiveness and elasticity to increase gel strength. However, within the 7.5–12.5% BFP range, heightened polysaccharide concentrations induce thermodynamic incompatibility with proteins, triggering phase separation and consequently reducing gel strength [19]. In contrast, at 15% BFP content, elevated polysaccharide levels facilitate a percolating colloidal network that reinforces the protein matrix through electrostatic screening effects, ultimately enhancing gel strength [20]. However, a BFP content of 15% darkened the sausage color, affecting its sensory quality.

As illustrated in Figure 2b, the gel strength of the vegan sausages initially increased and then decreased with CG addition. The maximum values were observed at 54% and 60%, reaching 300.02 ± 18.90 g·cm and 297.35 ± 25.71 g·cm, respectively, with no significant difference between them (*p* > 0.05). The addition of polysaccharide-based gelling enhancers, such as xanthan gum, konjac gum, and carrageenan, promotes tighter molecular alignment within the double helices in the gel network, improving the gel structure and increasing gel strength [21]. However, excessive CG competes with SPI for water molecules through its strong water-binding capacity, impeding continuous SPI network formation and consequently reducing gel strength while deteriorating textural quality [22].

As depicted in Figure 2c, the gel strength of the vegan sausages initially elevated and then declined with increasing content of gluten. At 35% gluten content, the gel strength peaked at 327.71 ± 11.99 g·cm, which was significantly higher than the other groups (*p* < 0.05). Gluten, composed of glutenin and gliadin, forms a robust three-dimensional network through hydration, providing structural support to the gel matrix of vegan sausages [23]. SPI, which is rich in hydrophilic carboxyl groups, forms a gel that interpenetrates the gluten network, resulting in a stable cross-linked gel system that enhances the textural properties of vegan sausages [24]. Therefore, moderate gluten incorporation improved the gel strength of meat analogs. However, at high gluten levels, it competed with SPI for water molecules, disrupting the formation of an integrated network. Owing to its higher water-binding affinity, gluten alters water distribution and induces proton exchange with SPI, ultimately compromising the gel’s structural integrity.

As demonstrated in Figure 2d, the gel strength of vegan sausages exhibited an initial sharp decline, followed by a steady downward trend as the water content increased from 300% to 550%. Within the appropriate range of addition, SPI and gluten formed a stable, gel-filled network structure with water, exhibiting strong gel properties. However, excessive water diluted the concentrations of SPI, gluten, and CG, resulting in reduced gel strength in vegan sausages [25]. Conversely, insufficient water content causes inadequate mixing of raw materials.

Based on a comprehensive gel strength analysis across all experimental variables, we determined the optimal formulation parameters of vegan sausages as follows: BFP, 5%; CG, 54%; gluten, 35%; and water, 350%.

### 3.2. Analysis of Orthogonal Experiment Results

Based on single-factor experiments, the concentrations of BFP (A), CG (C), and gluten (D) were chosen as independent variables. The formula was optimized using gel strength and sensory scores as evaluation indicators. The experimental results and variance analysis are presented in Table 4, respectively. According to the range analysis shown in Table 3, the impact of the three factors on gel strength of vegan sausages was ranked as follows: D > C > A, indicating that gluten had the greatest effect on the gel strength of vegan sausages, followed by CG, and BFP had the least effect. For sensory scores, the ranking was D > A > C, indicating that gluten had the greatest impact on the sensory scores, followed by BFP, whereas CG had the least effect. The optimal formula derived from the results for both gel strength and sensory scores was A_3_C_1_D_3_, which corresponded to 5% BFP, 56% CG, and 37% gluten.

Further analysis of variance was conducted using Fisher’s F-test to determine whether the factors significantly impacted the experimental parameters. A larger F-value indicates a more significant influence of the factors on the experimental indicators [14]. The variance analysis results (Table 5) showed that both gluten and CG content significantly affected the gel strength of the vegan sausages (*p* < 0.05). The gluten content significantly influenced both the gel strength and overall sensory quality of the vegan sausages (*p* < 0.05), whereas the CG content significantly affected the gel strength (*p* < 0.05). However, the amount of BFP only influenced the overall sensory quality without statistical significance (*p* > 0.05).

The optimal combination of the three factors identified by the orthogonal experiment was verified by reanalyzing two indicators. The values of gel strength and sensory score were 355.33 ± 3.29 g·cm and 9.30 ± 0.22, respectively, which were comparable to those of the same group (Group 8) in the orthogonal experiment and higher than other groups.

### 3.3. Physicochemical Properties Analysis of Key Factors

The ANOVA results showed that both gluten and CG content significantly affected the gel strength of BFP vegan sausages (*p* < 0.05). Consequently, a comprehensive analysis was conducted on the protein structure-function relationship, textural properties, microstructural network, and dynamic rheological behavior of gluten and CG at varying addition levels, which aimed to elucidate the gel formation and reinforcement mechanisms across multiple scales.

#### 3.3.1. Analysis of Free Sulfhydryl Groups, Total Sulfhydryl Groups, Disulfide Bonds, and Surface Hydrophobicity

The total TS consists of two parts: FS and those hidden within the protein structure. The S-S bonds formed by sulfhydryl groups serve as crucial covalent bonds in proteins, enhancing the compactness of the peptide chain. Sulfhydryl and S-S bonds can undergo interconversion via redox enzymes [26]. SH plays a crucial role in maintaining the stable conformation and biological activity of proteins. The SH content of vegan sausages reflects the number of hydrophobic amino acids on the protein surface, making it a common indicator for assessing changes in the protein molecular structure [27]. Since bromophenol blue binds to hydrophobic groups on protein surfaces, its binding amount can be used to characterize the SH content in the sample; the greater the binding of bromophenol blue, the higher the SH content [27]. Measuring FS, TS, S-S bonds, and SH in vegan sausage is a key analytical method for evaluating the molecular interactions in protein gels.

As exhibited in Table 6, the FS content followed a trend of first increasing and then decreasing as the GC content was raised (*p* < 0.05). In contrast, the TS content and number of S-S bonds first decreased significantly and then rebounded (*p* < 0.05). Additionally, the SH showed a significantly increasing trend (*p* < 0.05). The maximum FS content (54%) coincided with minimum values for TS (171 ± 0.61 mol/g) and S-S bonds (63.90 ± 0.39 mol/g), whereas SH (42.88 ± 0.39 µg) peaked at 56%. This trend might stem from the competition between the higher CG content and SPI for water binding, which reduced the water-holding capacity of SPI and promoted the interactions between proteins and water-dissolved oxygen molecules. Subsequently, the hydration was weakened, protein structures were altered, hydrogen bond networks and hydrophobic interactions were disrupted, and the hidden FS were exposed onto the molecular surface. Some sulfhydryls might oxidize to form new S-S bonds or covalently bind with other active components, which decelerated the sulfhydryl/S-S bond exchange reactions. Furthermore, the exposed FS engaged in oxidation or intermolecular crosslinking, promoting S-S bond formation, and re-exposing some previously hidden sulfhydryl groups [28].

As illustrated in Table 7, with increasing gluten content, the FS content exhibited a significant decline (*p* < 0.05), whereas the TS and S-S bond contents showed a significant upward trend (*p* < 0.05). This indicates that higher gluten levels promoted the conversion of FS groups into S-S bonds within the gel matrix. As a result, more protein molecules interacted with water, thereby enhancing hydration. Dissolved oxygen in water further accelerated the oxidation of FS groups into S-S bonds [19]. As gluten content increased, the SH of the vegan sausage initially increased but then declined, reaching a maximum value (46.70 ± 0.45 µg) at 35%. This trend might be attributed to enhanced exposure of hydrophobic groups on the protein surface by gluten addition. However, excessive protein aggregation can bury intrinsic proteins [29], resulting in lower SH in the 37% gluten group (42.85 ± 0.69 µg) than in the 35% gluten group.

#### 3.3.2. Texture Profile Analysis

The CG exhibits good gelling properties and plays a vital role in water retention, thickening, and stabilization during food processing [30]. As described in Table 8, with increasing CG content, the gel strength of the vegan sausages gradually increased, whereas the elasticity, cohesiveness, and resilience decreased, and chewiness varied. The vegan sausage with 56% CG demonstrated the highest gel strength (340.40 ± 21.95 g·cm), which was not significantly different from that of the 54% CG group (337.87 ± 32.43 g·cm) (*p* > 0.05). However, 56% group exhibited the lowest values for elasticity (0.43 ± 0.01 g·cm), cohesiveness (0.40 ± 0.05), and resilience (0.12 ± 0.00), whereas chewiness (1205.25 ± 80.24) was relatively high and showed no significant difference from the 52% group (1219.12 ± 196.24) (*p* > 0.05). This may be attributed to the ability of CG to form a three-dimensional network structure, enhance the crosslinking density, and promote gel formation [31]. At critical concentrations, a plateau phase may occur owing to phase separation or excessive crosslinking [32], while excessive addition may lead to the saturation of the interaction between the CG and proteins [33]. In addition, the water-holding capacity of the polysaccharides in the CG may soften the gel matrix [34]. Combined with Table 7, it can be inferred that the 54% CG group exhibited lower protein crosslinking and a weaker gel network, resulting in reduced chewiness.

The gel strength and texture parameters of the vegan sausages with different gluten contents are outlined in Table 9. As the gluten content elevated, the texture profile of the vegan sausages gradually improved. The highest values of the gel strength (327.88 ± 29.84 g·cm), springiness (0.56 ± 0.02), cohesiveness (0.44 ± 0.01), resilience (0.15 ± 0.01), and chewiness (1308.61 ± 29.28) were achieved at 37% gluten addition. This indicates that a higher gluten content positively improved the texture of the vegan sausage, showing a linear enhancement with increasing gluten levels. This phenomenon may be attributed to the fact that glutenin and gliadin in gluten can form a robust gluten network structure upon hydration, promoting gel formation as their content increases [29].

#### 3.3.3. Microstructure Analysis

**Figure 3:**
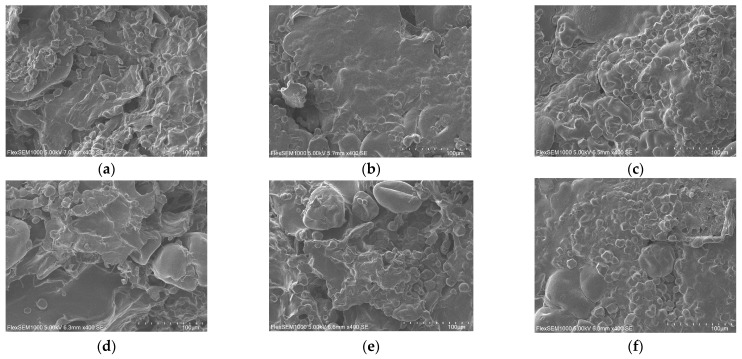
Effects of different addition rates of CG and gluten on the microstructure of BFP vegan sausage. Notes: (**a**), (**b**), and (**c**) represent 52%, 54%, and 56% CG, respectively, and (**d**), (**e**), and (**f**) represent 33%, 35%, and 37% gluten, respectively.

The microstructures of the gel systems with varying concentrations of CG and gluten, magnified 400× under SEM, are shown in Figure 3. Among the different CG groups, the 52% CG group exhibited the largest pores and an irregular network structure with both dense and loose regions. This non-uniformity likely resulted from inadequate CG content to form a homogeneous gel structure. This morphology is unfavorable for water retention, leading to a lower water-holding capacity of the samples [26]. In contrast, both the 54% and 56% CG groups displayed uniform and compact gel structures, indicating superior gel performance, a finding corroborated by the gel strength measurements. This occurs because the increased CG content enhances the overall gel structure of the vegan sausage by forming its own hydrophilic colloidal network and interacting with the protein network, thereby filling and reinforcing the structure [35]. Consequently, this improves textural properties such as gel strength and elasticity.

As the gluten content increased, the gel structure of the vegan sausages became more compact with finer pores. This is because higher gluten content facilitates the oxidation of free sulfhydryl groups into disulfide bonds, promoting protein cross-linking and dense structure formation. Additionally, gluten binds free water within the gel structure and swells to fill structural voids [36], effectively improving structural integrity. Consequently, textural properties such as gel strength and elasticity are enhanced. At 37% gluten content, the porous gel structure of the vegan sausage became uniformly fine-textured and stable. This modification resulted in improvements in the water-holding capacity and gel strength, which align with the measured gel strength results.

#### 3.3.4. UV-Visible Spectroscopy Analysis

**Figure 4:**
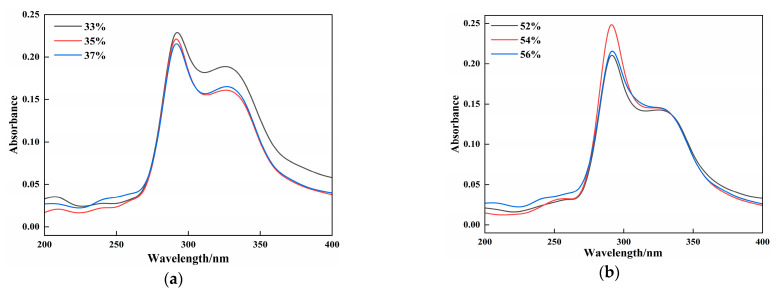
Effects of different additions of gluten (**a**) and CG (**b**) on the UV absorption characteristics of BFP vegan sausage.

UV-visible absorption spectroscopy reflects protein structural changes and protein–small molecule interactions in samples. Previews studies have elucidated that the interaction strength affects the peak intensity changes, whereas peak shifts indicate microenvironmental alterations around the chromophores due to these interactions [37]. As shown in Figure 4, the absorption intensities varied among the vegan sausages with different gluten and CG contents; however, their spectral shapes, peak emergence points, and peak positions remained similar. All groups exhibited absorption peaks at 290 cm^−1^ and 330 cm^−1^. The absorption peaks between 250 and 290 cm^−1^ were attributed to the benzene ring structures of phenylalanine, tyrosine, and tryptophan in proteins, whereas the peak at 330 cm^−1^ was speculated to originate from the oil phase in vegan sausages. In Figure 4a, the intensities of the UV-visible absorption peaks decreased with increasing gluten content. The absorption peaks of the 35% and 37% gluten groups were similar, indicating that a higher gluten powder content promoted crosslinking protein in the samples, leading to the embedding of some hydrophobic bonds in the aromatic heterocycles responsible for the absorption peaks, thus reducing the peak intensity. This phenomenon is linked to structural alterations in the protein. Protein cross-linking can alter secondary structure, modifying the microenvironment of aromatic amino acid residues and consequently affecting their absorbance at specific wavelengths. Furthermore, cross-linking may change protein conformation, leading to either exposure or burial of hydrophobic regions, thereby influencing their physicochemical properties. Figure 4b illustrates that as the CG content increased, the UV-visible absorption peak intensity first increased and then decreased. The 54% CG group exhibited the highest absorption peak intensity, whereas the 52% and 56% CG groups showed similar peaks. This may be attributed to CG altering the polar environment of the sample, causing the molecular chains to unfold and exposing more hydrophobic groups in the aromatic heterocycles, thus increasing the absorption intensity. However, excessive CG introduces steric hindrance, embedding some proteins and resulting in reduced peak intensity [26].

#### 3.3.5. Dynamic Rheological Properties Analysis

**Figure 5:**
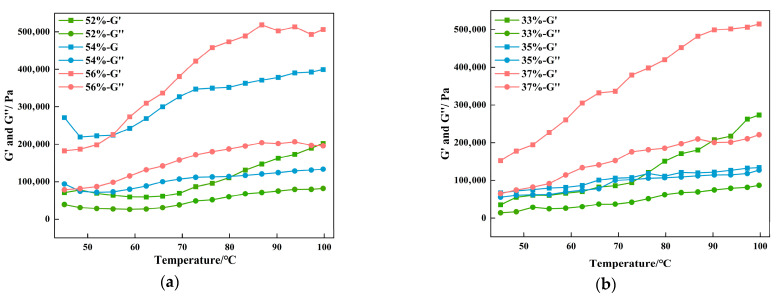
Variation curves of storage modulus (G′) and loss modulus (G″) of BFP vegan sausage with different amounts of CG (**a**) and gluten (**b**).

The G′ and G″ of the CG (Figure 5a) and gluten groups (Figure 5b) both exhibited an upward trend as the temperature rose. G′ was significantly higher than G″ throughout the process without any intersection, indicating that the vegan sausages, regardless of their CG and gluten content, demonstrated significant elastic characteristics, with the elastic modulus remaining dominant. During heating, the rate of protein gel formation increases, along with enhanced crosslinking of protein polymers, leading to the development of a network structure [38]. The 56% CG and 37% gluten groups established the steepest curve slopes, indicating the highest gelation rates, shortest time required to reach stability, and ultimately the highest gel strength. In general, gluten forms a stable gel network through strong intermolecular forces, whereas CG, despite its relatively weak intermolecular interactions, has a higher water-binding capacity, enabling it to form a gel network structure [39,40].

## 4. Conclusions

This study optimized the formulation process of BFP vegan sausages using single-factor experiments and orthogonal tests. The effects of BFP, gluten, and CG content on the overall sensory quality and gel characteristics of vegan sausages were explored, and the underlying mechanisms linking these key factors to the gel structure and functionality were clarified. The results showed that the optimal formulations (component/SPI mass ratio, %) were 5% BFP, 56% CG, 37% gluten, 40% corn starch, 13% sugar, 8% salt, 8% yeast extract, 8% I + G, 8% soybean oil, with water added at 350% (*w*/*w*, SPI basis). Oneway-ANOVA analysis indicated that both gluten and CG significantly affected the gel strength of vegan sausages (*p* < 0.05), while gluten also significantly influenced the sensory quality (*p* < 0.05). As the gluten content increased, the FS content decreased significantly (*p* < 0.05), whereas the TS content and S-S bonds rose significantly (*p* < 0.05). In contrast, increasing the CG content initially increased the FS levels and then decreased, whereas the TS content and S-S bonds exhibited the opposite trend. The SH level also elevated significantly (*p* < 0.05). The gel strength of the vegan sausage improved with higher gluten and CG content, peaking at 37% gluten and 56% CG. SEM observations confirmed a uniform and dense gel network, further validating the positive effect of these proportions on gel structure. These suggested that gluten and CG enhanced the gel properties of vegan sausages through distinct mechanisms: gluten primarily strengthened the gel performance via intermolecular disulfide crosslinking and hydrophobic group exposure, whereas CG improved the gel structure through physical filling. The findings of this study establish a scientific basis for utilizing BFP as a sustainable protein source in premium plant-based meat alternatives. Notably, BFP extracts exhibit antioxidant, moisturizing, and antimicrobial activities. These compounds may serve as natural preservatives, humectants, or functional ingredients in plant-based foods, though their extraction processes, safety profiles, and industrial-scale application require further investigation.

## Figures and Tables

**Table 1:** Single factor experiment table with four factors (component/SPI mass ratio, %).

No.	BFP	CG	Gluten	Water
1	5.0	48	18	300
2	7.5	54	24	330
3	10.0	60	30	360
4	12.5	66	36	390
5	15.0	72	42	420
6	-	78	-	-

Note: The other components were kept constant as follows: corn starch (40%), white sugar (13%), salt (8%), yeast extract (8%), I + G (8%), and soybean oil (8%).

**Table 2:** Influencing factors and levels of L_9_(3^4^) orthogonal experiment.

No.	Factors (Component/SPI Mass Ratio, %)			
	A (BFP)	B	C (CG)	D (Gluten)
1	1 (6)	2	1 (56)	1 (35)
2	1	1	2 (52)	2 (33)
3	1	3	3 (54)	3 (37)
4	2 (4)	2	2	3
5	2	1	3	1
6	2	3	1	2
7	3 (5)	2	3	2
8	3	1	1	3
9	3	3	2	1

Note: The other components were kept constant as follows: corn starch (40%), white sugar (13%), salt (8%), yeast extract (8%), I + G (8%), and soybean oil (8%) with water added at 350% (*w*/*w*, SPI basis).

**Table 3:** Sensory evaluation scoring criteria for BFP vegan sausage.

Color (1–2)	Hardness, Elasticity (4.5–6)	Morphology (0.5–2)
Red, uniform color (1.5–2)	Suitable hardness, toughness, and elasticity (5.5–6)	No pores observed in cross-section (1.5–2)
Deep red, uniform color (1–1.5)	Slight hardness, toughness, and elasticity (5–5.5)	Few pores in the cross-section (1–1.5)
Dark brown, dull surface (0.5–1)	Too hard or too soft (4.5–5)	Numerous pores in cross-section (0.5–1)

**Table 4:** Range analysis (R) was performed on the two indicators obtained from the orthogonal experiment.

Indicators (i)		Factors (j)				Gel Strength (g·cm)	Sensory Score
		A (BFP)	B	C (CG)	D (Gluten)		
1		1	2	1	1	187.99 ± 16.42	7.90 ± 0.17
2		1	1	2	2	204.09 ± 23.54	8.10 ± 0.31
3		1	3	3	3	275.41 ± 17.57	8.40 ± 0.34
4		2	2	2	3	284.76 ± 19.92	9.00 ± 0.30
5		2	1	3	1	154.50 ± 8.88	8.20 ± 0.23
6		2	3	1	2	247.03 ± 18.30	8.80 ± 0.20
7		3	2	3	2	217.61 ± 27.75	8.60 ± 0.15
8		3	1	1	3	361.15 ± 25.20	9.30 ± 0.29
9		3	3	2	1	201.20 ± 32.98	8.10 ± 0.32
Gel strength (g·cm)	k_1j_	222.50	230.53	265.39	181.20		
	k_2j_	229.14	239.88	230.43	222.91		
	k_3j_	259.99	241.22	215.81	307.52		
	R	37.49	10.69	49.58	126.32		
	Best level	A_3_	B_3_	C_1_	D_3_		
Sensory score	k_1j_	8.13	8.50	8.67	8.07		
	k_2j_	8.67	8.53	8.40	8.50		
	k_3j_	8.67	8.43	8.40	8.90		
	R	0.53	0.10	0.27	0.83		
	Best level	A_3_	B_2_	C_1_	D_3_		

**Table 5:** Analysis of variance (ANOVA) results.

	Factors	Sum of Squares of Deviations	Degrees of Freedom	F-Ratio	F-Critical Value	Significance
Gel strength	A	2401.54	2	11.81	19.00	
	B	203.41	2	1.00	19.00	
	C	3894.45	2	19.14	19.00	*
	D	24,855.15	2	122.19	19.00	*
	Error	203.41	2			
Sensory score	A	0.57	2	35.56	19.00	*
	B	0.02	2	1.00	19.00	
	C	0.14	2	8.88	19.00	
	D	1.04	2	65.13	19.00	*
	Error	0.02	2			

Note: * indicates a significant difference (*p* < 0.05).

**Table 6:** Effects of different CG additions on free sulfhydryl groups, total sulfhydryl groups, disulfide bonds, and surface hydrophobicity of BFP-based vegan sausage.

CG Content/%	FS (mol/g)	TS (mol/g)	S-S Bonds (mol/g)	SH (µg)
52	28.42 ± 0.21 ^b^	242.63 ± 0.45 ^b^	107.10 ± 0.33 ^b^	24.78 ± 0.42 ^c^
54	43.26 ± 0.19 ^a^	171.06 ± 0.61 ^c^	63.90 ± 0.39 ^c^	37.15 ± 0.67 ^b^
56	21.03 ± 0.04 ^c^	264.92 ± 1.38 ^a^	121.95 ± 0.58 ^a^	42.88 ± 0.66 ^a^

Different letters in the same column indicate significant differences among the groups (*p* < 0.05).

**Table 7:** Effects of different gluten additions on free sulfhydryl groups, total sulfhydryl groups, disulfide bonds, and surface hydrophobicity of BFP-based vegan sausage.

Gluten Content/%	FS (mol/g)	TS (mol/g)	S-S Bonds (mol/g)	SH (µg)
33	35.83 ± 0.45 ^a^	117.33 ± 0.49 ^c^	40.75 ± 0.49 ^c^	42.70 ± 0.57 ^b^
35	22.44 ± 0.68 ^b^	174.14 ± 1.44 ^b^	75.85 ± 0.84 ^b^	46.70 ± 0.45 ^a^
37	21.03 ± 0.25 ^c^	263.71 ± 0.49 ^a^	121.34 ± 0.30 ^a^	42.85 ± 0.69 ^b^

Different letters in the same column indicate significant differences among the groups (*p* < 0.05).

**Table 8:** Effect of different CG additions on the textural properties of BFP vegan sausage.

CG Content/%	Gel Strength (g·cm)	Springiness	Cohesiveness	Resilience	Chewiness
52	295.13 ± 9.45 ^b^	0.49 ± 0.01 ^a^	0.43 ± 0.01 ^a^	0.13 ± 0.00 ^a^	1219.12 ± 196.24 ^a^
54	337.87 ± 32.43 ^a^	0.49 ± 0.00 ^a^	0.44 ± 0.01 ^a^	0.14 ± 0.00 ^a^	954.93 ± 74.65 ^b^
56	340.40 ± 21.95 ^a^	0.43 ± 0.01 ^b^	0.40 ± 0.05 ^b^	0.12 ± 0.00 ^b^	1205.25 ± 80.24 ^a^

Different letters in the same column indicate significant differences among the groups (*p* < 0.05).

**Table 9:** Effects of different gluten additions on the textural properties of BFP-based vegan sausage.

Gluten Content/%	Gel Strength (g·cm)	Springiness	Cohesiveness	Resilience	Chewiness
33	256.98 ± 8.42 ^b^	0.50 ± 0.01 ^c^	0.44 ± 0.00 ^b^	0.13 ± 0.00 ^b^	837.92 ± 82.09 ^c^
35	278.52 ± 30.30 ^ab^	0.54 ± 0.02 ^b^	0.44 ± 0.01 ^b^	0.13 ± 0.00 ^b^	954.11 ± 73.12 ^b^
37	327.88 ± 29.84 ^a^	0.56 ± 0.00 ^a^	0.44 ± 0.00 ^a^	0.15 ± 0.00 ^a^	1308.61 ± 29.28 ^a^

Different letters in the same column indicate significant differences among the groups (*p* < 0.05).

## Data Availability

The data presented in this study are available on request from the corresponding author due to privacy concerns.

## Abbreviations

The following abbreviations are used in this manuscript:

ANOVA	Analysis of variance
BFP	*Bangia fusco-purpurea*
CG	Composite gel
S-S	Disulfide bonds
FS	Free sulfhydryl
PBS	Phosphate-buffered saline
SEM	Scanning electron microscopy
SH	Surface hydrophobicity
SPI	Soybean protein isolate
TPA	Texture profile analysis
TS	Total sulfhydryl
